# Prognostic value of soluble PD-L1 and exosomal PD-L1 in advanced gastric cancer patients receiving systemic chemotherapy

**DOI:** 10.1038/s41598-023-33128-9

**Published:** 2023-04-28

**Authors:** Kabsoo Shin, Joori Kim, Se Jun Park, Myung Ah Lee, Jae Myung Park, Myung-Gyu Choi, Donghoon Kang, Kyo Young Song, Han Hong Lee, Ho Seok Seo, Sung Hak Lee, Bohyun Kim, Okran Kim, Juyeon Park, Nahyeon Kang, In-Ho Kim

**Affiliations:** 1 Division of Medical Oncology, Department of Internal Medicine, Seoul St. Mary’s Hospital, College of Medicine, The Catholic University of Korea, 222 Banpo-daero, Seocho-gu, Seoul, 06591 South Korea; 2 Division of Gastroenterology, Department of Internal Medicine, Seoul St. Mary’s Hospital, College of Medicine, The Catholic University of Korea, Seoul, South Korea; 3 Department of Surgery, Seoul St. Mary’s Hospital, College of Medicine, The Catholic University of Korea, Seoul, South Korea; 4 Department of Clinical Pathology, Seoul St. Mary’s Hospital, College of Medicine, The Catholic University of Korea, Seoul, South Korea; 5 Department of Radiology, Seoul St. Mary’s Hospital, College of Medicine, The Catholic University of Korea, Seoul, South Korea; 6 Cancer Research Institute, College of Medicine, The Catholic University of Korea, Seoul, South Korea; 7 Department of Gastric Cancer Centre, Seoul St. Mary’s Hospital, College of Medicine, The Catholic University of Korea, Seoul, South Korea

**Keywords:** Prognostic markers, Gastric cancer

## Abstract

The prognostic role of soluble PD-L1 (sPD-L1) and exosomal PD-L1 (exoPD-L1) in patients with gastric cancer (GC) receiving systemic chemotherapy remains unelucidated. Thus, we examined their prognostic significance in patients with advanced GC. Blood samples were obtained from 99 patients with advanced GC receiving first-line chemotherapy. Serum-derived exosomes were isolated by centrifugation and polymer precipitation. The correlation between serum-derived exoPD-L1, plasma sPD-L1, immune-related markers, and circulating immune cells was evaluated. Patients were divided into two groups according to pretreatment sPD-L1 and exoPD-L1 levels: low sPD-L1 and high sPD-L1 groups, low exoPD-L1 and high exoPD-L1 groups. Patients with low sPD-L1 level before treatment (< 9.32 pg/mL) showed significantly better overall survival (OS) and progression-free survival (PFS) than those with high sPD-L1 level (≥ 9.32 pg/mL). The low exoPD-L1 group (< 10.21 pg/mL) showed a tendency of longer PFS than the high exoPD-L1 group (≥ 10.21 pg/mL). Pretreatment sPD-L1 was an independent prognostic factor for OS in multivariate analysis. exoPD-L1 was associated with systemic inflammation markers, immunomodulatory cytokines, and T cells, while sPD-L1 was associated with tumor markers. Pretreatment plasma-derived sPD-L1 level could be used as a prognostic marker for patients receiving cytotoxic chemotherapy. Serum-derived exoPD-L1 may reflect the immunosuppressive state of patients with advanced GC.

## Introduction

Gastric cancer (GC) is the fifth most common cancer and the fourth leading cause of cancer-related deaths worldwide; it is especially prevalent in Eastern Asia and Eastern Europe^1^. Systemic chemotherapy is the mainstay treatment for advanced GC; however, the prognosis is poor^2–5^. Recently, the combination of the programmed death-1 (PD-1) inhibitor, nivolumab, with cytotoxic chemotherapy has shown survival benefits in unresectable advanced or metastatic GC and has become the standard first-line treatment for patients with a PD-L1 combined positive score (CPS) of 5 or higher^6^.

PD-L1 is a membrane-bound ligand found mainly in tumor cells and microenvironmental immune cells. When PD-L1 binds to the PD-1 receptor, an immune checkpoint receptor is expressed on T cells, and it transduces an inhibitory signal that inhibits T cell activation^7^. The PD-L1/PD-1 axis has been found to be an important mechanism for tumor cells to evade host immunity and has been identified as a major target of cancer immunotherapy using immune checkpoint inhibitors^7–9^. The degree of PD-L1 expression in tumor and tumor microenvironment can be measured using tumor proportion score (TPS) and CPS. TPS measures PD-L1 expression in tumor cells only, while CPS measures the total number of PD-L1-positive cells, including both tumor cells and immune cells in the tumor microenvironment.

Many studies have been conducted to investigate the relationship between PD-L1 expression in tumor tissues and prognosis in different cancers^10–15^. Several clinical trials have found that PD-L1 expression in the tumor and tumor microenvironment has a predictive value for response to PD-1 inhibitors^16–20^. However, the temporal and spatial heterogeneity of tissue PD-L1 expression limits its prognostic and predictive value^21^. To address this limitation, recent studies have focused on the extracellular forms of PD-L1.

PD-L1 can be found in multiple extracellular forms, such as microvesicles, exosomes, and secreted or cleaved forms in the blood. Soluble PD-L1 (sPD-L1) is an extracellular form of PD-L1 that can be detected in the blood, and recent studies have shown that soluble PD-L1 retaining PD-1 binding domain could bind to the PD-1 receptor of immune cells to negatively regulate their antitumor immune response^22^. Several studies have been conducted to investigate the clinical utility of sPD-L1 in various cancers^23–30^.

Exosomes are nano-sized, lipid bilayer membrane-bound extracellular vesicles formed by the endocytic pathway and secreted following the fusion of multiple vesicular bodies with the plasma membrane. Exosomes contain proteins, lipids, and nucleic acids of the cells of origin, which are considered important mediators of intercellular communication^31^. Exosomal PD-L1 (exoPD-L1) derived from tumor suppresses T cells and higher levels of exoPD-L1 have been associated with poor survival in different types of tumors^32–34^. exoPD-L1 has been found to be a potential biomarker for recurrence in postoperative GC patients, and it is associated with the prognosis of GC because of its potential immunosuppressive role^35,36^.

However, the prognostic role of both sPD-L1 and exoPD-L1 in patients with advanced gastric cancer receiving chemotherapy has not been thoroughly investigated. The current study aimed to investigate the prognostic and predictive significance of both sPD-L1 and exoPD-L1 in patients with advanced GC receiving first-line chemotherapy.

## Results

### Clinical characteristics

A total of 99 patients were recruited for analysis between March 2019 and March 2021. The median OS was 12.4 months (95% CI 10.4–15.2 months). Table 1 summarizes the patient baseline characteristics; 89 patients with HER2-negative cancer received first line fluoropyrimidine/platinum chemotherapy (XELOX [n = 57], FOLFOX [n = 24], SP [n = 2], XP [n = 6]), and 10 patients with HER2-positive cancer received trastuzumab and XP (n = 7) or FP (n = 3). Twenty-two patients had recurrence after curative surgery, and 77 were initially metastatic or had advanced GC. Microsatellite instability (MSI) status was as follows: MSI tumor (n = 5), microsatellite stable tumor (n = 86), and undetermined (n = 8). EBV (Epstein-Barr virus) status was as follows: EBV-positive (n = 6), EBV-negative (n = 87), and undetermined (n = 6).

**Table 1:** Baseline characteristics.

Variables		Low	High		Low	High	
		*sPD-L1*	*sPD-L1*		*exoPD-L1*	*exoPD-L1*	
	n = 99	n = 72	n = 27	*p* value*	n = 38	n = 61	*p* value*
Age (years)	Range (37–83)			0.059			0.208
< 65	**52 (**52.5%)	**42 (**58.3%)	**10 (**37.0%)		**23 (**60.5%)	**29 (**47.5%)	
≥ 65	**47 (**47.5%)	**30 (**41.7%)	**17 (**63.0%)		**15 (**39.5%)	**32 (**52.5%)	
Sex				0.149			0.079
Male	**70 (**70.7%)	**48 (**66.7%)	**22 (**81.5%)		**23 (**60.5%)	**47 (**77.0%)	
Female	**29 (**29.3%)	**24 (**33.3%)	**5 (**18.5%)		**15 (**39.5%)	**14 (**23.0%)	
ECOG PS				0.701			0.729
0, 1	**90 (**90.9%)	**66 (**91.7%)	**24 (**88.9%)		**34 (**89.5%)	**56 (**91.8%)	
2	**9 (**9.1%)	**6 (**8.3%)	**3 (**11.1%)		**4 (**10.5%)	**5 (**8.2%)	
Differentiation				0.113			0.343
Well to moderate	**26 (**26.3%)	**22 (**30.6%)	**4 (**14.8%)		**12 (**31.6%)	**14 (**23.0%)	
Poor	**73 (**73.7%)	**50 (**69.4%)	**23 (**85.2%)		**26 (**68.4%)	**47 (**77.0%)	
HER2				0.341			0.912
Positive	**10 (**10.1%)	**6 (**8.3%)	**4 (**14.8%)		**4 (**10.5%)	**6 (**9.8%)	
Negative	**89 (**89.9%)	**66 (**91.7%)	**23 (**85.2%)		**34 (**89.5%)	**55 (**90.2%)	
Disease status				0.058			0.476
Locally advanced	**9 (**9.1%)	**9 (**12.5%)	**0 (**0.0%)		**2 (**5.3%)	**7 (**11.5%)	
Recurrent or metastatic	**90 (**90.9%)	**63 (**87.5%)	**27 (**100.0%)		**36 (**94.7%)	**54 (**88.5%)	
Peritoneal seeding				0.837			0.130
Yes	**53 (**53.5%)	**39 (**54.2%)	**14 (**51.9%)		**24 (**63.2%)	**29 (**47.5%)	
No	**46 (**46.5%)	**33 (**45.8%)	**13 (**48.1%)		**14 (**36.8%)	**32 (**52.5%)	
No. of metastatic sites				**0.036**			0.293
≥ 2	**35 (**35.4%)	**21 (**29.2%)	**14 (**51.9%)		**11 (**28.9%)	**24 (**39.3%)	
< 2	**64 (**64.6%)	**51 (**70.8%)	**13 (**48.1%)		**27 (**71.1%)	**37 (**60.7%)	
CEA (ng/mL)				**0.007**			0.349
> 5	**31 (**31.3%)	**17 (**23.6%)	**14 (**51.9%)		**14 (**36.8%)	**17 (**27.9%)	
≤ 5	**68 (**68.7%)	**55 (**76.4%)	**13 (**48.1%)		**24 (**63.2%)	**44 (**72.1%)	
CA 19-9 (U/mL)				0.492			0.535
> 37	**35 (**35.4%)	**24 (**33.3%)	**11 (**40.7%)		**12 (**31.6%)	**23 (**37.7%)	
≤ 37	**64 (**64.6%)	**48 (**66.7%)	**16 (**59.3%)		**26 (**68.4%)	**38 (**62.3%)	
Tissue PD-L1				0.877			0.058
CPS ≥ 10	**44 (**44.4%)	**31 (**43.1%)	**13 (**48.1%)		**21 (**55.3%)	**23 (**37.7%)	
CPS < 10	**46 (**46.5%)	**34 (**45.8%)	**12 (**44.4%)		**12 (**31.6%)	**34 (**55.7%)	
Undetermined	**9 (**9.1%)	**7 (**9.7%)	**2 (**7.4%)		**5 (**13.2%)	**4 (**6.6%)	

Data are n (%). *p value from Chi-square test or Fisher's exact test for categorical variables.

*HER2* human epidermal growth factor receptor 2, *CEA* carcinoembryonic antigen, *CA 19-9* Cancer antigen 19-9, *PD-L1* programmed death ligand 1, *CPS* combined positive score.

Significant *p*-values < 0.05 are shown in bold.

### Determination of optimal cutoff value of pretreatment exoPD-L1 and sPD-L1

The median exoPD-L1 level for all patients was 11.90 pg/mL (range: 0.02–69.87 pg/mL). The median plasma sPD-L1 level was 5.62 pg/mL (range: 0.12–23.93 pg/mL). To determine optimal cut-off values of exoPD-L1 and sPD-L1 levels, maximally selected log-rank statistics was applied to discriminate the patients into two groups in terms of OS^37^. The cut-off values of exoPD-L1 and sPD-L1 were 10.21 pg/mL and 9.32 pg/mL, respectively.

Based on the cutoff value of exoPD-L1, patients were grouped into the high exoPD-L1 group (≥ 10.21 pg/mL; 61 (61.6%)) and low exoPD-L1 group (< 10.21 pg/mL; 38 (38.4%)). Based on the cutoff value of sPD-L1, patients were grouped into the high sPD-L1 group (≥ 9.32 pg/mL; 27 (27.3%)) and low sPD-L1 group (< 9.32 pg/mL; 72 (72.7%)).

### Correlations among exoPD-L1, sPD-L1, and tissue PD-L1

The correlations of exoPD-L1 levels with sPD-L1 and tissue PD-L1 were analyzed in 90 patients whose tissue PD-L1 CPS was obtained. No correlation was found between exoPD-L1 and sPD-L1. No correlation was found between extracellular PD-L1 and tissue PD-L1 CPS. There was a weak correlation between sPD-L1 and CPS in the 44 patients with CPS ≥ 10 (Spearman’s **ρ** = 0.306, *p* = 0.044) (Supplementary Fig. S1 online).

### Prognostic value of exoPD-L1 and sPD-L1

**Figure 1:**
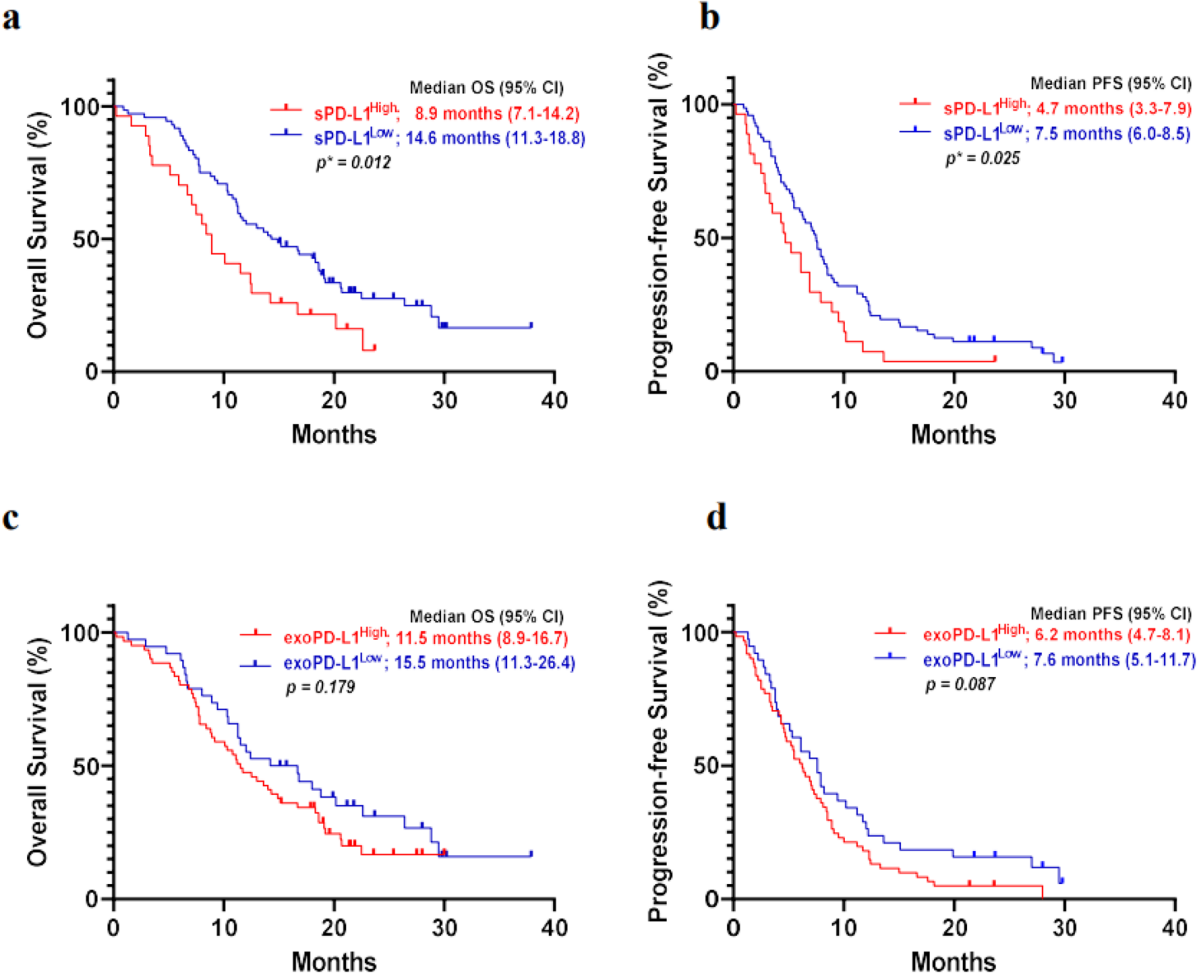
Survival between soluble and exoPD-L1 high and low groups. (**a**,**b**) OS and PFS in the low sPD-L1 group was longer than that in the high sPD-L1 group (**c**,**d**). OS and PFS were not different between the exoPD-L1 groups.

The low sPD-L1 group showed longer OS than the high sPD-L1 group (OS: 14.6 months vs. 8.9 months, *p* = 0.012). The low sPD-L1 group also showed longer PFS than the high sPD-L1 group (PFS: 7.5 months vs. 4.7 months, *p* = 0.025) (Fig. 1). OS and PFS were longer in the low exoPD-L1 group but not significantly different (OS: 15.5 months vs. 11.5 months, *p* = 0.179, PFS: 7.6 months vs. 6.2 months, *p* = 0.087) (Fig. 1).

### Level of exoPD-L1 and sPD-L1 according to clinical response

**Figure 2:**
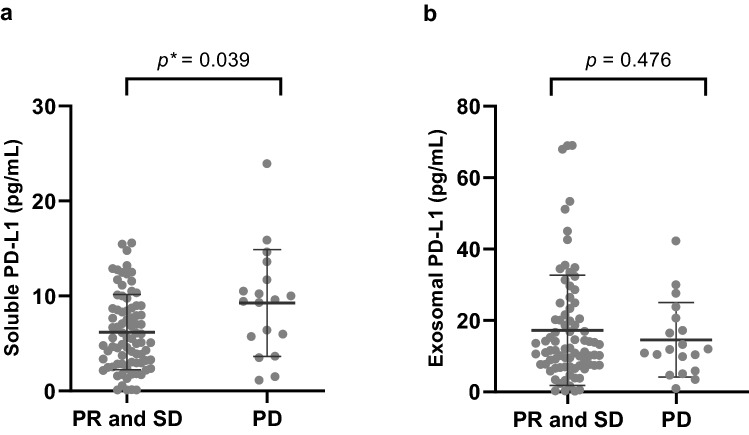
Level of exoPD-L1 and sPD-L1 according to clinical response (**a**) baseline sPD-L1 was significantly lower in partial response and stable disease (PR and SD) group than progressive disease (PD) group (**b**) the level of exoPD-L1 was not different between the groups. Unpaired *t*-test was performed between two groups.

The best overall response was partial response (PR) in 42 patients (42.4%), stable disease (SD) in 38 patients (38.4%), progressive disease (PD) in 17 patients (17.2%), and not evaluable in two patients (2.0%). Pretreatment sPD-L1 was higher in the PD group than in the PR and SD group (*p* = 0.039). But, pretreatment exoPD-L1 was not different between PD group and PR and SD group. (Fig. 2).

### Correlation with systemic inflammation markers and tumor markers

**Figure 3:**
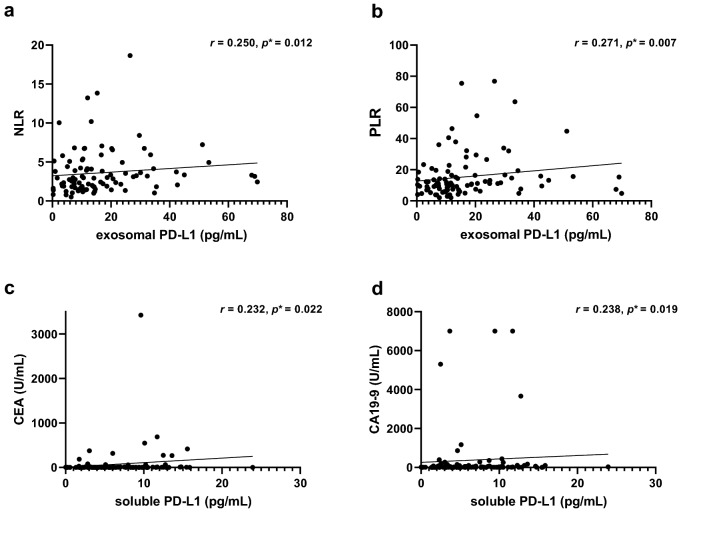
Correlation between exoPD-L1 and systemic inflammatory makers. (**a**,**b**) exoPD-L1 was positively correlated with NLR (Spearman’s **ρ** = 0.250, *p* = 0.012) and PLR (Spearman’s **ρ** = 0.271, *p* = 0.007). Correlation between sPD-L1 and tumor markers. (**c**,**d**) sPD-L1 was positively correlated with CEA (Spearman’s **ρ** = 0.232, *p* = 0.022) and CA 19-9 (Spearman’s **ρ** = 0.238, *p* = 0.019).

The correlation between both extracellular PD-L1 and systemic inflammatory markers was investigated. Systemic inflammation markers included white cell count (WBC), absolute neutrophil count (ANC), absolute lymphocyte counts (ALC), platelet count, neutrophil to lymphocyte ratio (NLR), and platelet to lymphocyte ratio (PLR). The NLR (Spearman’s **ρ** = 0.250, *p* = 0.012) and PLR (Spearman’s **ρ** = 0.271, *p* = 0.007) were both positively correlated with exoPD-L1, and ALC (Spearman’s **ρ** = − 0.231, *p* = 0.021) was negatively correlated with exoPD-L1 (Fig. 3, Supplementary Fig. S2 online). However, sPD-L1 was not correlated with any inflammation markers above.

The correlation with tumor markers was also investigated. Both CEA and CA 19-9 showed a weak positive correlation with sPD-L1 level (sPD-L1 and CEA; Spearman’s **ρ** = 0.232, *p* = 0.022, sPD-L1 and CA 19-9; Spearman’s **ρ** = 0.238, *p* = 0.019) (Fig. 3), but exoPD-L1 was not correlated with both tumor markers.

### Correlation with immunomodulatory cytokines

**Figure 4:**
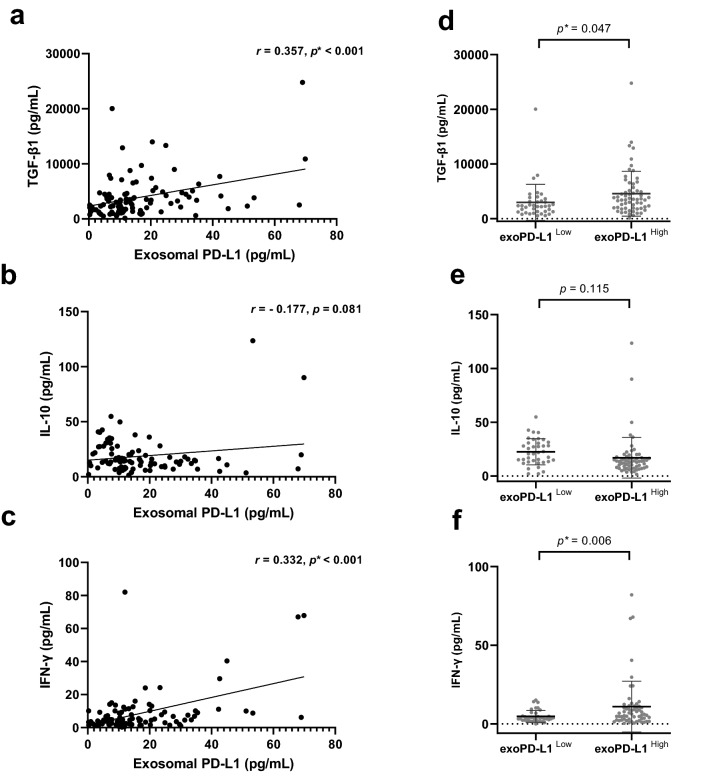
Correlation between baseline exoPD-L1 and cytokines. (**a**,**c**) exoPD-L1 showed a positive correlation with TGF-β1 and IFN-**γ**. (**b**) exoPD-L1 tended to negatively correlate with IL-10. (**d**,**f**) a higher level of TGF-β1 and IFN-**γ** was found in the high exoPD-L1 group than in the low exoPD-L1 group. (**e**) IL-10 level was higher in low exoPD-L1 group without significance. Spearman’s correlation analysis was performed in (**a**–**c**) Unpaired *t*-test was performed between two groups (**d**–**f**).

The correlation of both sPD-L1 and exoPD-L1 with immunomodulatory cytokines was explored. exoPD-L1 was positively correlated with TGF-β1 (Spearman’s **ρ** = 0.357, *p* < 0.001) and IFN-**γ** (Spearman’s **ρ** = 0.332, *p* < 0.001). The high exoPD-L1 group had higher levels of TGF-β1 and IFN-**γ** than the low exoPD-L1 group (TGF-β1; *p* = 0.047, IFN-**γ**; *p* = 0.006) (Fig. 4). However, sPD-L1 was not associated with the cytokines investigated.

### Correlation with T lymphocytes

**Figure 5:**
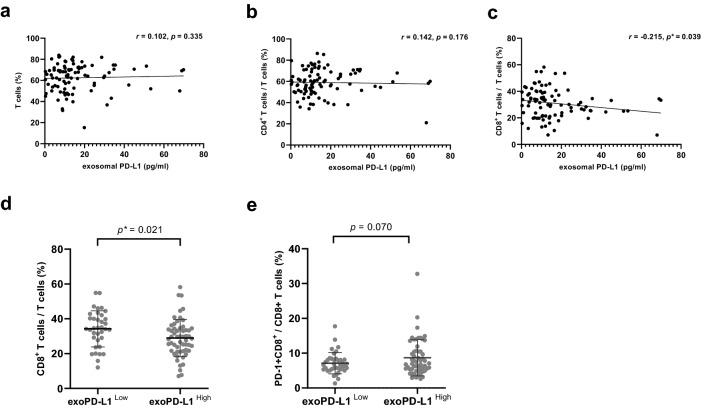
Correlation between types of T cells and exoPD-L1. (**a**–**c**) The correlation between exoPD-L1 level and the frequency of total T cells (**a**), CD4^+^ T cells (**b**), and CD8^+^ T cells (**c**). (**d**) The low exoPD-L1 group showed a higher frequency of CD8^+^ T cells than the high exoPD-L1 group. (**e**) The high exoPD-L1 group showed a tendency to be higher frequency of PD-1^+^CD8^+^ T cells. (*p* = 0.070) Unpaired *t*-test was performed between two groups.

The correlation between extracellular PD-L1 and peripheral T lymphocyte was analyzed. exoPD-L1 level was negatively correlated with the frequency of CD8^+^ T cells in peripheral blood (Spearman’s **ρ** = − 0.215, *p* = 0.039). The high exoPD-L1 group showed a significantly lower frequency of CD8^+^ T cells than the low exoPD-L1 group (*p* = 0.021). The high exoPD-L1 group showed a higher frequency of PD-1^+^CD8^+^ T cells in CD8^+^ T cells than the low exoPD-L1 group (Fig. 5). No correlation was observed between exoPD-L1 level and the frequency of T cells or CD4^+^ T cells. No correlation was observed between sPD-L1 and T cells.

### Univariate and multivariate analyses of survival

In univariate analysis, HER2 (positive), CA 19-9 (> 37 U/mL), number of metastatic sites (≥ 2), high sPD-L1, and high NLR were prognostic factors associated with OS. After adjusting for covariates, multivariate analysis showed that the number of metastatic sites (≥ 2) (HR 1.20, 95% CI 1.06–1.35, *p* = 0.004), high sPD-L1 (HR 1.97, 95% CI 1.18–3.28, *p* = 0.009), and high NLR (HR 1.67, 95% CI 1.00–2.79, *p* = 0.048) were independent prognostic factors for OS (Table 2).

**Table 2:** Univariate and multivariate analyses for overall survival.

	Univariate			Multivariate		
	HR	(95% CI)	*p* value	HR	(95% CI)	*p* value
Overall survival						
Age (≥ 65)	1.15	0.74–1.78	0.539			
Sex (male)	1.15	0.70–1.91	0.580			
ECOG PS (2)	1.36	0.70–1.91	0.105			
Differentiation (poor)	1.46	0.84–2.54	0.177			
HER2 (positive)	2.40	1.13–5.09	**0.023**	1.31	0.59–2.92	0.503
Disease status (metastatic)	1.61	0.70–3.71	0.264			
Peritoneal metastasis (present)	1.37	0.87–2.17	0.176			
No. of metastatic sites (≥ 2)	1.23	1.10–1.39	**< 0.001**	1.20	1.06–1.35	**0.004**
CEA (> 5 ng/mL)	0.86	0.52–1.42	0.551			
CA 19-9 (> 37 U/mL)	1.49	0.93–2.38	0.095	1.21	0.73–1.99	0.468
NLR (≥ 2.84)	2.19	1.38–3.47	**< 0.001**	1.67	1.00–2.79	**0.048**
PLR (≥ 150)	1.12	0.72–1.77	0.612			
Tissue PD-L1 (CPS ≥ 10)	0.90	0.56–1.44	0.653			
exoPD-L1 (high)	1.41	0.88–2.26	0.157			
sPD-L1 (high)	1.88	1.15–3.10	**0.013**	1.97	1.18–3.28	**0.009**

Significant *p*-values < 0.05 are shown in bold.

## Discussion

In this prospective study, we showed that a higher level of pretreatment sPD-L1 was associated with worse OS and PFS in advanced GC patients receiving first-line cytotoxic chemotherapy. We also showed that sPD-L1 was associated with a more advanced disease and that exoPD-L1 was associated with immunomodulatory cytokines and inflammatory markers.

Previous studies have explored the clinical implications of sPD-L1 as a prognostic marker of GC. They showed that a higher level of sPD-L1 was associated with a poor prognosis^26–28^. However, the retrospective design and relatively small number of patients with advanced-stage disease limited the implications of the findings. The prospective cohort in our study included nine patients with locally advanced/unresectable GC and 90 patients with metastatic GC. All nine patients with locally advanced GC were categorized into the low sPD-L1 group, and the high sPD-L1 group had a higher proportion of patients with more than two metastatic sites than did the low sPD-L1 group. sPD-L1 was also positively correlated with the tumor markers, CEA and CA 19-9. Considering that these tumor markers are associated with a more advanced disease in GC, a higher level of sPD-L1 might indicate a more advanced disease^38^ This could be one of the reasons why sPD-L1 could be a potential prognostic factor. In multivariate analysis, sPD-L1 showed a higher prognostic significance than any other factors, including CEA and CA 19-9.

Among the different forms of extracellular PD-L1, exoPD-L1 was considered to be a more potent immunosuppressant due to its structural stability and the presence of other molecules related to T cell signaling, such as MHC-I^35^. Fan et al. found that serum exoPD-L1 was associated with immunosuppression in GC patients by assessing the correlation between exoPD-L1 and immunosuppressive cytokines, and exoPD-L1 and circulating T cells^35^. In our study, exoPD-L1 found to have a positive correlation with TGF-β1 and high exoPD-L1 group showed lower frequency of CD8^+^T cells than low exoPD-L1 group, consistent with the study of Fan et al.^35^ Moreover, the frequency of PD-1^+^CD8^+^ T cells in CD8^+^ T cells tended to be higher in high exoPD-L1 group. Considering interaction between PD-L1 including exoPD-L1 and PD-1 receptor on PD-1^+^CD8^+^ T cells might result in dysfunction and exhaustion of the T cells and enhanced TGF-β signaling in tumor microenvironment, higher level of PD-1^+^CD8^+^ T cells in high exoPD-L1 group might also indicate immunosuppressive status of the disease^39^. Kuan et al. recently published a study that showed PD-1^+^CD8^+^ T cells abundance in the tumor microenvironment is associated with T-cell exhaustion and enhanced TGF-β signaling, and predicts poor prognosis in stage I-III gastric cancer^40^. Saito et al. also showed that circulating PD-1^+^CD8^+^ T cells may predict the prognosis of patients with stage I-III gastric cancer^41^. Combining these findings, high levels of exoPD-L1 may indicate an immunosuppressive status, which can contribute to poor prognosis in gastric cancer, although our cohort did not show statistically significant results.

Interestingly, exoPD-L1 also showed a positive correlation with IFN-γ, which is related to activated T cells. The complex results of exoPD-L1 showing a positive correlation with both TGF-β and IFN-γ may reflect the diverse effects of systemic cytokines that can be influenced by various factors in the body. Moreover, this correlation could be interpreted in the context that chronic inflammation induced by cancer reflects the immunosuppressive environment^42^. We also found that exoPD-L1 was positively correlated with the systemic inflammatory markers, NLR and PLR. However, sPD-L1 was not correlated with immunomodulatory cytokines and inflammatory markers.

Several studies have compared the prognostic value of sPD-L1 and exoPD-L1 in GC. Fan et al. showed that exoPD-L1 had a better prognostic value than serum sPD-L1 in GC. Li et al. also showed that exoPD-L1 could be more prognostic by comparing exoPD-L1 with sPD-L1 in post-operative GC patients^35,36^. Both studies suggested that the better prognostic value of exoPD-L1 might be due to its immunosuppressive role. In our cohort, the prognostic value of exoPD-L1 was not superior to that of sPD-L1. This conflicting result might be due to the more advanced disease status of the patients in our cohort, which included only advanced/unresectable and metastatic GC. The study by Fan et al. included only 31 patients (40%) of stage IV out of 69 patients and Li’s study included about 27 patients (9%) of stage IV out of 331 patients. This indicates that the prognostic value of exoPD-L1 could be more significant in earlier GC than in advanced GC. In addition, subsequent PD-1 inhibitor treatment might lead to insignificant survival difference between exoPD-L1 high and low group, although predictive role of exoPD-L1 was not established in advanced gastric cancer patients receiving PD-1 inhibitors.

The origin of circulating PD-L1 remains unclear and could be derived from multiple sources, including tumor cells and surrounding immune cells. The correlation between tissue PD-L1 expression and circulating PD-L1 has been investigated in previous studies; however, the results are controversial. Several studies on lung cancer, renal cell carcinoma, and melanoma have shown that there is no correlation between sPD-L1 and tissue PD-L1^43–45^. A study on 180 patients who underwent surgery for gastric cancer also analyzed the relationship between sPD-L1 and tissue PD-L1, but no correlation was found^46^. However, recent studies have revealed a correlation between sPD-L1 and tissue PD-L1 expression in colorectal cancer and hepatocellular carcinoma^29,47^. These controversial results are similarly observed for exoPD-L1, wherein studies on lung cancer and melanoma have shown a positive correlation between exoPD-L1 and tissue PD-L1^48,49^, whereas others have not found a correlation^50,51^. The reasons for these controversial results could be the unclear source of extracellular PD-L1, the influence of the spatial and temporal heterogeneity of tissue PD-L1, and the difference in methods used to measure PD-L1 expression in tissue and blood. In our study, no correlation was observed between tissue PD-L1 and sPD-L1 in all patients or between the latter and exoPD-L1. However, sPD-L1 was positively associated with CPS in the high-PD-L1 expression group (CPS ≥ 10). This might indicate that patients with high CPS may have a higher proportion of sPD-L1 derived from both the tumor and the tumor microenvironment compared to other unclear sources. Thus, it is possible that the correlation between CPS and sPD-L1 may be more likely to be observed in patients with high CPS. In future studies, to elucidate the relationship between tissue PD-L1 expression and extracellular PD-L1, CPS, which measures the expression of PD-L1 in both tumor and immune cells within the tumor microenvironment, would be a more appropriate option for evaluating PD-L1 expression in tissue compared to solely measuring PD-L1 expression in cancer cells.

In our study, exoPD-L1 did not show a clear correlation with sPD-L1, which is similar to the results of previous studies on head and neck cancer and colorectal cancer^29,32^. This lack of a correlation may suggest that they serve different biological roles. In our study, both exoPD-L1 and sPD-L1 showed correlations with different factors. Considering that sPD-L1 showed potential prognostic value for advanced GC and that exoPD-L1 might be associated with the host immunosuppressive status, further research into the prognostic and predictive value of both sPD-L1 and exoPD-L1 for patients with GC receiving PD-1 inhibitors alone or in combination with cytotoxic chemotherapy is required.

### Limitations

In addition to the pretreatment level of sPD-L1 and exoPD-L1, dynamic changes in sPD-L1 and exoPD-L1 also need to be investigated to further understand the role of both forms of PD-L1; however, this was not investigated herein. Moreover, the tissue PD-L1 status of nine patients was not investigated mostly due to insufficient samples, which might have affected the results of the correlation between tissue PD-L1 and liquid PD-L1.

In addition, as described in the “Methods” section, three models were compared to define optimal cut-off of circulating PD-L1, and the model using maximally selected rank statistics was selected because the cut-off by the model demonstrated the highest statistical significance. Thus, the analysis in this study was based on the arbitrary cut-off determined by this model. To address the issue of this arbitrary cut-off, the correlation between extracellular PD-L1 and immunological factors and other systemic markers was thoroughly analyzed in the entire cohort, regardless of the cut-off value. Significant correlations were found, suggesting that exoPD-L1 reflects the immunosuppressive status of the host and that sPD-L1 is related to a more advanced disease status. When an analysis was further performed according to high- and low- PD-L1 groups divided by the cut-off, the correlations were still present, with a significant difference between the groups identified via a t-test. As a result, the cut-off value effectively supported the main hypothesis of the study, specifically that exoPD-L1 reflects the immunosuppressive status of the host. These efforts could have partially addressed the limitation of the arbitrary cut-off value, but caution should still be exercised when interpreting the results.

In this study, patients were divided into groups based on an arbitrary cut-off of exoPD-L1 and sPD-L1, and these were analyzed as categorical values, which showed statistical significance between sPD-L1 groups. However, when analyzed as continuous values, similar trends were shown, but statistically significant results were not obtained. The number of patients in this study (n = 99) may not have been sufficient to indicate statistical power as continuous values for sPD-L1 or exoPD-L1. Nevertheless, when sPD-L1 was analyzed in a multivariate model as a categorical value, its statistical significance remained along with the significance of other factors known to be associated with prognosis. Further research with more patients is needed to validate the clinical significance of extracellular PD-L1.

## Conclusion

The level of pretreatment sPD-L1 was associated with a more advanced disease in advanced GC and could be used as a prognostic marker for patients receiving systemic chemotherapy. Serum-derived exoPD-L1 may reflect the immunosuppressive state of patients with advanced GC. Further investigation is required to determine the prognostic and predictive role of both sPD-L1 and exoPD-L1 in patients receiving immune check point inhibitors.

## Materials and methods

### Patients

We prospectively enrolled patients with untreated GC at the Catholic University of Seoul St. Mary’s Hospital between March 2019 and March 2021. The eligibility criteria for the study were as follows: (1) ≥ 20 years old; (2) histologically confirmed gastric adenocarcinoma; (3) unresectable advanced or recurrent/metastatic disease; (4) scheduled to receive first-line systemic chemotherapy; (5) provision of written informed consent.

The chemotherapy regimens used were fluorouracil and platinum doublet, 5-FU, oxaliplatin, and leucovorin (FOLFOX), capecitabine and oxaliplatin (XELOX), 5-FU and cisplatin (FP), S-1 and cisplatin (SP), and capecitabine and cisplatin (XP). Trastuzumab was added to the treatment regimen for human epidermal growth factor receptor 2 (HER2)-positive GC. Blood samples were collected before and after the chemotherapy.

The study was approved by the Institutional Review Board of the Catholic University of Seoul Saint Mary’s Hospital (KC18TNSI0361). All patients signed a written informed consent before enrollment.

### Extraction and verification of exosome

The ExoQuick™ Exosome Precipitation Solution kit (System Biosciences, Palo Alto, CA, USA) was used to isolate serum-derived exosomes according to the manufacturer’s instructions. In brief, 500 µL of serum were added to 120 µL of exosome precipitation solution and slowly mixed and incubated for 1 h at 4℃ and centrifuged at 1,500 × g for 30 min. The supernatant was discarded and exosome pellet was precipitated at the bottom. The resulting pellet was resuspended into 250µL of sterile phosphate buffered saline (PBS) and stored at -80 °C until ready for use. Transmission Electron Microscopy (TEM) was used to confirm the isolated exosomes. For TEM, samples were freshly isolated and diluted in nuclease-free water. After fixation in 4% paraformaldehyde for 1 h, 5uL of exosome sample on grid were analyzed by TEM using a JEM 1010 transmission electron microscope (JEOL, Tokyo, Japan) equipped with a Gatan Orius SC1000 CCD camera (Gatan, Pleasanton, California, USA). The representative TEM image of the verified exosomes can be found in Supplementary Fig. S3 online.

### ELISA

Plasma samples were centrifuged at 2,500 g for 10 min and the supernatants were stored at -80 ˚C until further usage. Plasma levels of Transforming growth factor β1 (TGF-β1), TGF-β2, Ki-67, granzyme B, Interleukin-10 (IL-10), Interferon-** γ** (IFN-**γ)**, and PD-L1 were measured using ELISA kits (R & D Systems, Minneapolis, MN, USA), following the manufacturer’s instructions. For exoPD-L1 ELISA analysis, exosomes were isolated from 500 µL of serum and the exosome pellet was dissolved in 250 µL of PBS prior to use in experiments. The PD-L1 levels in extracted exosomes (exoPD-L1) were measured using the same method as described above. Information about antibodies used in ELISA is in Supplementary Table S1.

### Tissue PD-L1 and combined positive score (CPS)

PD-L1 immunohistochemistry assays were performed using 22C3 pharmDx (Dako, Santa Clara, CA, USA) on the Dako Autostainer Link 48 with EnVision DAB Detection System (Agilent Technologies, Santa Clara, CA, USA), according to the manufacturer’s instructions.

The CPS was calculated by dividing the total number of PD-L1-positive cells (tumor cells, lymphocytes, and macrophages) by the total number of tumor cells and multiplying the result by 100.

$${\text{CPS}} = \frac{{{\text{PD}} - {\text{L}}1\;{\text{positive}}\;{\text{cells}} \left( {{\text{Tumor}}\;{\text{cells}},\;{\text{lymphocytes}},\;{\text{and}}\;{\text{macrophage}}} \right) \times 100}}{{{\text{viable}}\;{\text{tumor}}\;{\text{cells}}}}$$

### FACS analysis

Peripheral blood mononuclear cells (PBMCs) from patients were isolated from Ficoll-Paque density-centered blood that had been treated with EDTA and stored in liquid nitrogen before analysis. Markers used for thawing and staining PBMCs included CD4, CD8, CD69, CD45, CD3, and PD-1(Biolegend, San Diego, CA, USA). The stained cells were acquired using BD Biosciences Canto II (BD Biosciences, San Diego, CA, USA), and the data were analyzed using FlowJo software (Tree Star, Ashland, OR, USA). Information about antibodies used in FACS is in Supplementary Table S1.

### Statistical analysis

The time from the start of treatment to disease progression or death from any cause was defined as progression-free survival (PFS). The time from the start of treatment to death from any cause or the last follow-up date was defined as overall survival (OS). Treatment response was evaluated using the Response Evaluation Criteria in Solid Tumors version 1.1.

Maximally selected rank statistics and a time-dependent receiver operating characteristic curve were used to determine the optimal cutoff value of exoPD-L1 and other clinical values, which were compared with the median value. The chi-squared and Fisher’s tests for categorical variables were used to compare the clinicopathological differences between the groups.

Univariate analyses for OS and PFS were performed using the Kaplan–Meier method and the log-rank test. Cox proportional hazards regression analyses were used for multivariate models to verify the prognostic values of clinical variables, including exoPD-L1 and sPD-L1. Statistical significance was set at *p* < 0.05. All statistical analyses were performed using R version 4.1.1 (http://www.r-project.org).

### Human rights statement and informed consent

All procedures followed were in accordance with the ethical standards of the responsible committee on human experimentation (institutional and national) and with the Helsinki Declaration of 1964 and later versions. Informed consent to be included in the study, or the equivalent, was obtained from all patients.

## Supplementary Information

Supplementary Information.

## Data Availability

The data used and/or analyzed during the study are available from the corresponding author on reasonable request.
