# Supplementary material for: Prognostic value of soluble PD-L1 and exosomal PD-L1 in advanced gastric cancer patients receiving systemic chemotherapy
Source: Sci Rep. 2023 Apr 28;13:6952. doi: 10.1038/s41598-023-33128-9 (PMC10147600; doi:10.1038/s41598-023-33128-9)
Supplement: Supplementary file 1 — Supplementary Information. [file 41598_2023_33128_MOESM1_ESM.pdf]

Supplementary Figures and Tables

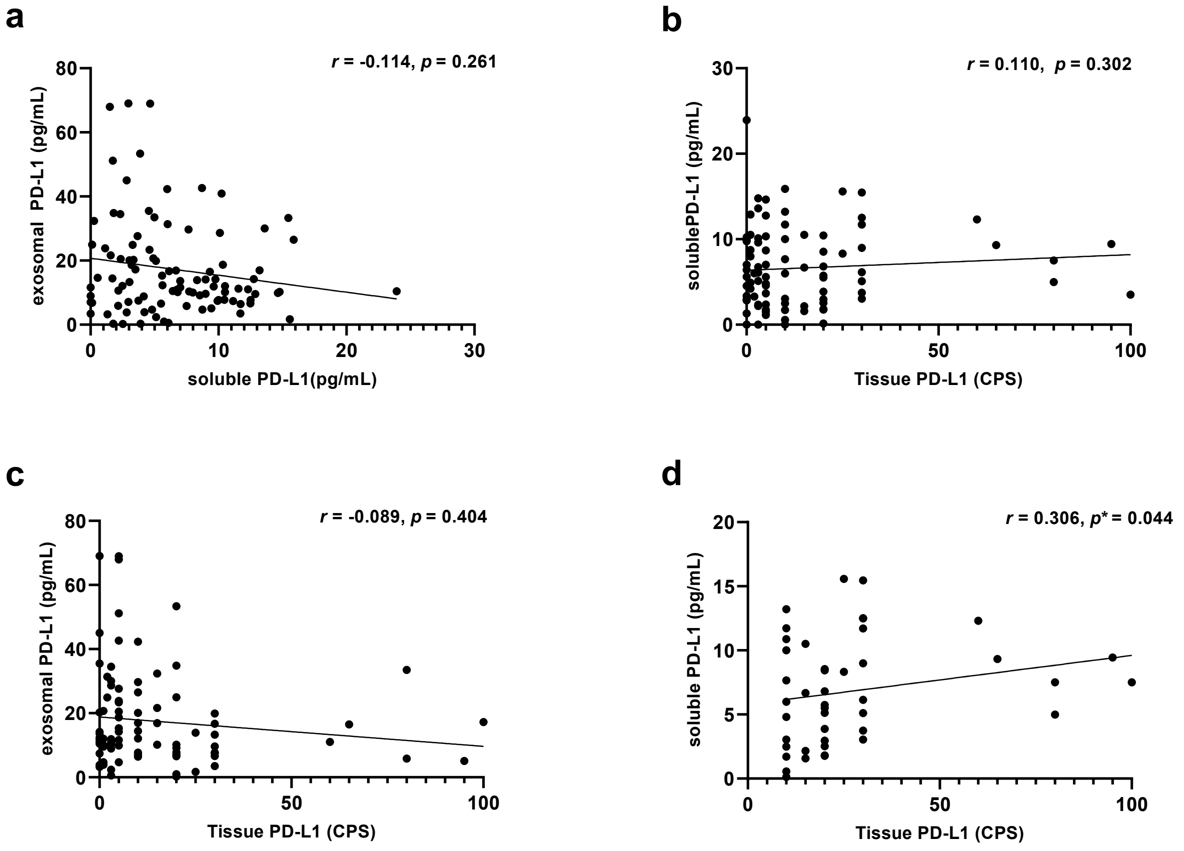

Fig. S1 Correlation between extracellular PD-L1 and tissue PD-L1 (a) both sPD-L1 and exoPD-L1 showed no correlation (b, c) CPS and both extracellular PD-L1 showed no correlation. (d) CPS and sPD-L1 showed a weak correlation in patients with CPS $\geq$ 10 (Spearman's ρ = 0.306, *p* = 0.044). Statistical analysis was performed using Spearman correlation. *CPS : combined positive Score*

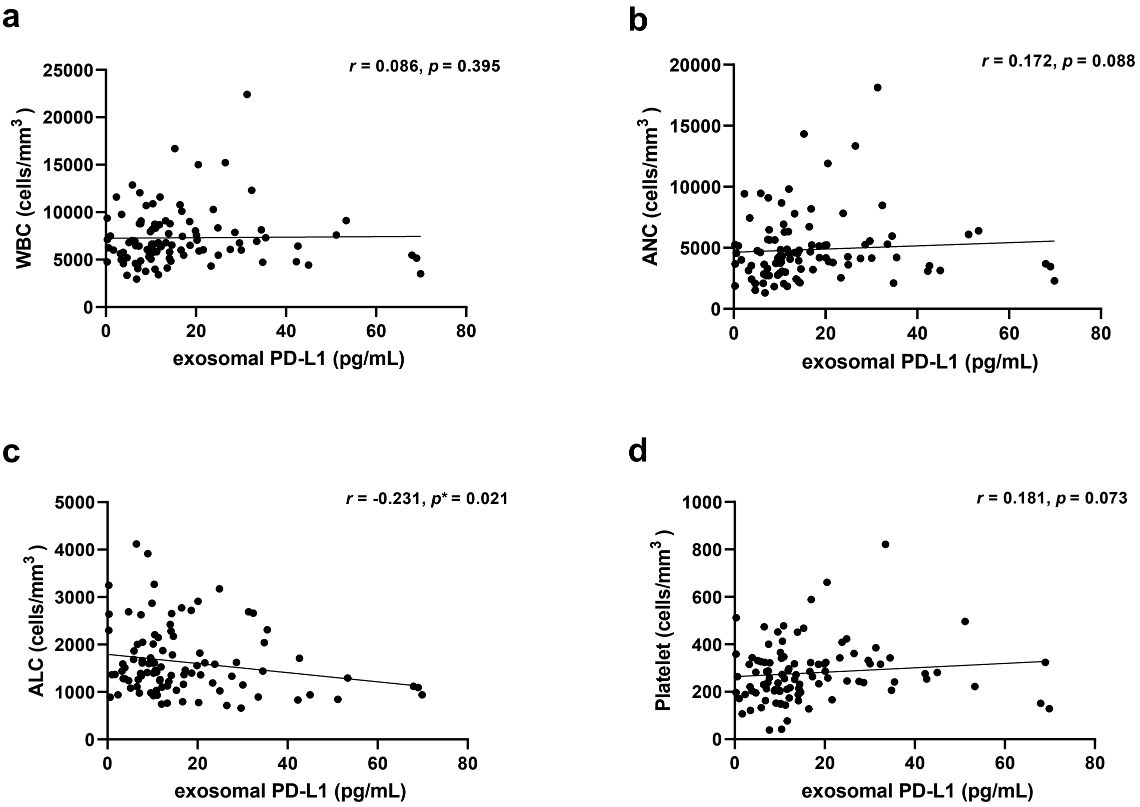

Fig. S2 Correlation between exoPD-L1 and white blood cell (WBC), absolute neutrophil count (ANC), absolute lymphocyte count (ALC), and platelet. **(**a, b, c, d**)** exoPD-L1 was tending to be correlated with ANC and platelet. exoPD-L1 was negatively correlated with ALC (Spearman's r = -0.231, *p* = 0.021). Statistical analysis was performed using Spearman correlation.

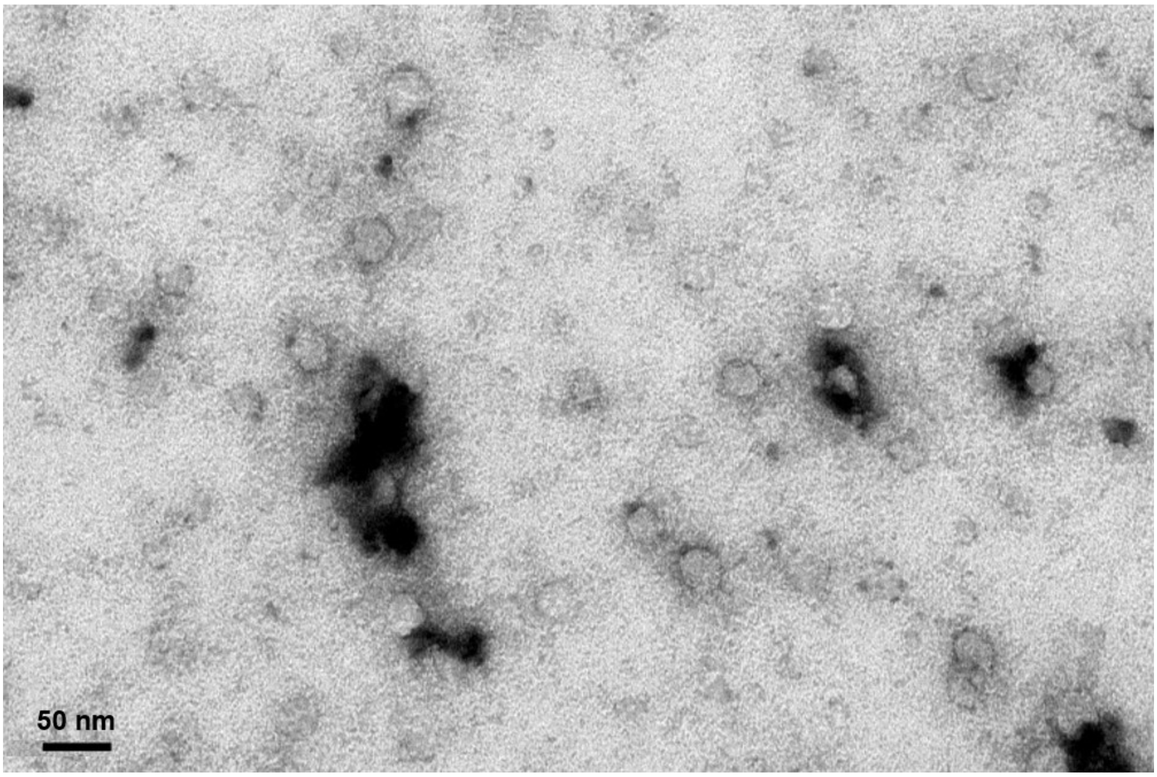

Fig. S3 Representative electron microscopy image of isolated serum exosomes from patients with advanced gastric cancer. Transmission Electron Microscopy (TEM) verified less than 50nm sized sphere-shaped vesicles with bilayer.

Supplementary Table S1

Antibody	Supplier	Cat. No.
**FACS**		
CD4	BioLegend	357416
CD8	BioLegend	300910
CD69	BioLegend	310912
CD45	BioLegend	368510
CD3	BioLegend	317314
PD-1	BioLegend	329908
**ELISA**		
TGF-beta1	R & D Systems	DY240
TGF-beta2	R & D Systems	DY3020
Ki-67	R & D Systems	DY7617-052
GranzymeB	R & D Systems	DY2906-05
IL-10	R & D Systems	DY217B-05
IFN-γ	R & D Systems	DY285B-05
PD-L1	R & D Systems	DY156
**Exosome markers**		
CD63	Santa Cruz Biotechnology	Sc-5275
TSG101	Santa Cruz Biotechnology	Sc-7964
